# Research on the Hydrodynamic Performance of Manta Rays Using a 2D CFD Model

**DOI:** 10.3390/biomimetics10060348

**Published:** 2025-05-26

**Authors:** Wenxian Li, Kai Ni, Cunjun Li, Chaoqiang Nan, Shijie Su

**Affiliations:** 1 Zhoushan Institute of Calibration and Testing for Quality and Technology Supervision, Zhoushan 316021, China; 231110201111@stu.just.edu.cn; 2 School of Mechanical Engineering, Jiangsu University of Science and Technology, Zhenjiang 212100, China; 231210201414@stu.just.edu.cn (K.N.); 231110201112@stu.just.edu.cn (C.N.)

**Keywords:** kinematics, quasi-propulsive efficiency, propulsive force, manta ray pectoral fins, fluctuating motion

## Abstract

Currently, the most commonly used method to study the hydrodynamic performance of manta rays is computational fluid dynamics (CFD) simulation. In this research, we investigated the effects of kinematic parameters—specifically wave number, amplitude, and frequency—on the hydrodynamic performance of manta rays during the swimming process by constructing a 2D CFD model. First, we verified the reasonableness of the 2D simulation. Subsequently, a 2D simulation was used to study the hydrodynamic performance of manta ray pectoral fins, and it was concluded that using low-amplitude, high-frequency propulsion with an optimal wave number has better energy utilization. Finally, we conducted orthogonal experiments, which revealed that the thrust reaches a maximum value of 8.55 N at a frequency of 1 Hz, amplitude of 0.3 c, and wave number of 0.4, and the quasi-propulsive efficiency reaches a maximum value of 82.4% at a frequency of 0.8 Hz, amplitude of 0.3 c, and wave number of 0.4. In general, we can regulate the wave number to a range of 0.35 to 0.4, the frequency to between 0.7 and 0.9 Hz, and the amplitude to between 0.3 c and 0.325 c. This configuration yields a thrust exceeding 3.04 N and a quasi-propulsive efficiency surpassing 70.4%.

## 1. Introduction

Studies on bionic fish have demonstrated the high efficiency, stability, and excellent stealth of the pectoral fin-propelled bionic fish in the median and/or paired fin propulsion (MPF) mode [1,2]. As a representative of the MPF mode, manta rays use their pectoral fins on both sides to flutter to obtain thrust and realize straight forward swimming, steering, and lateral tilting.

Currently, numerous researchers are conducting studies on the hydrodynamic performance of bionic fish using CFD techniques. Studies on bionic fish can be categorized into two main types: replacing the fish body or fins with 2D models and emphasizing the realistic modeling of fish. Abbaspoor et al. [3] utilized the NACA0012 airfoil cross-section to examine the characteristics and performance of pairs of airfoil propulsive vortices in two significant natural mechanisms: pure pitch and fluctuating oscillations. Anderson et al. [4] experimentally explored the impact of pitch and droop combinations on the generation of thrust under constrained spans and large motion amplitudes. Dong et al. [5,6] summarized the effects of spreading ratio, Strahl number, and Reynolds number on the wake topology and airfoil performance at very low Reynolds numbers of Re = 100, 200, 400. Monnier et al. [7,8] conducted pitching experiments to illustrate the importance of oscillation frequency and amplitude on wake structure, thrust coefficient, and vortex–vortex interaction.

To further investigate the hydrodynamic performance of manta ray pectoral fins, researchers have conducted detailed modeling and numerical simulations of the fin’s structure and kinematics. Giovanni Bianchi et al. [9] investigated how the frequency and wavelength of pectoral fin motions influence thrust, power, and velocity. Their findings indicated that velocity increases with frequency and has a maximum for a specific wavelength, while efficiency depends solely on the wavelength. Gao et al. [10,11,12,13] investigated manta rays using a sphere function-based gas kinetic scheme (SGKS) combined with an immersed boundary method. Their findings indicated that manta rays achieve maximum thrust at a wave number of 0.4. Additionally, the propulsive efficiency initially increases with amplitude before decreasing. Hadi Safari et al. [14] investigated the effect of kinematic parameters such as Strouhal number, non-dimensional fluctuation amplitude ($h^{*}$), the maximum angle of attack ($\alpha_{0}$), and oscillation velocity on the hydrodynamic performance of manta rays during the swimming process. Their results indicated that geometric and kinematic parameters, such as phase lag between heave and pitch, heave amplitude, and maximum angle of attack, have a significant effect on thrust, power consumption, and efficiency.

Numerous parameters influence the hydrodynamic performance of pectoral fins. Typically, multiple kinematic parameters act synergistically to affect this performance; however, many studies isolate and vary only one parameter while fixing the others. Consequently, it is essential to examine the simultaneous effects of three key kinematic parameters—amplitude, frequency, and wave number—on manta rays’ hydrodynamics. In this study, a 2D CFD model was employed to comprehensively analyze the impact of these parameters on manta ray propulsion.

This paper is organized as follows: First, we present the modeling of manta ray pectoral fins and relevant validation. Next, we demonstrate the rationality of using a 2D CFD model to investigate the hydrodynamic performance of manta rays through experimental methods. We also examine the effects of wave number, amplitude, and frequency on the hydrodynamic performance of manta rays. Additionally, we conduct orthogonal experiments to explore the intervals of enhanced swimming propulsion and efficiency in manta rays. Finally, we present the conclusions of this study.

## 2. Materials and Methods

### 2.1. Physical Modeling of Manta Ray Pectoral Fins

As in previous studies, the roughness of the pronotum, tail, and body surface was not considered in this work. Since the primary focus of this study was to evaluate the effects of various hydrodynamic parameters on the fluctuating propulsion performance of manta rays under dynamic conditions, these parameters can be neglected under certain circumstances. For example, Clark et al. [15] focused on the swimming patterns of their subjects and modeled only the caudal fin for caudal fin-propelled fish. Similarly, Chen and Wu [16,17] ignored the shapes of the anal and head regions in their study of manta rays, while Chen et al. [18] replaced the true pectoral fin shapes with triangular approximations in their research of cow nose rays. To reduce computational time, the mesh is simplified while preserving essential geometric features and leveraging the symmetry of the paired pectoral fins during straight forward swimming; all other features are omitted. Consequently, only a unilateral pectoral fin is modeled.

**Figure 1:**
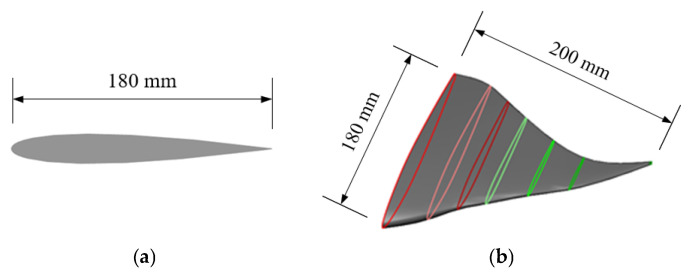
Physical model of pectoral fins of manta rays: (**a**) 2D pectoral fin outline; (**b**) 3D model of pectoral fins of manta rays (colored ovals are pectoral fin cross-section outline).

In this paper, we modeled the NACA0012 airfoil as a 2D fish body profile with a chord length of 200 mm and a spread length of 180 mm (Figure 1). The 3D manta ray pectoral fins were subsequently constructed using multisegmented release and multistrip control methods. The contours of each profile were derived by integrating shape data from the NACA airfoil coordinate database [19]. A 2D airfoil was then generated using Profili 2 airfoil design software and imported into SolidWorks (version 2022) to create a 3D surface via multisegmented release and multistrip control methods.

### 2.2. Numerical Methods and Mesh-Independent Validation

In this work, the overlapping mesh technique and the user-defined function (UDF) module [20] in Fluent 2020R2 were employed to simulate both 2D models and 3D models. The simulation was conducted using an incompressible hydrodynamic model, the turbulence model utilized was SST k-omega, and the coupling of pressure and velocity in the continuum equations was implemented using the complied algorithm for the 2D model and the simple algorithm for the 3D model, with the convergence residuals set to $1\times 10^{-3}$. Boundary conditions were defined as follows: the inlet boundary was set as the velocity inlet, the outlet boundary was set as the pressure outlet, and the perimeter was a fixed wall. The UDF module primarily used the dynamic mesh model macro DEFINE_MESH_MOTION to define fluctuations on the surface of the pectoral fins, and it utilized the COMPUTE_FORCE_AND_MOMENT macro to calculate the force in the x-direction of the pectoral fins at each time step and convert it into a linear velocity, which was assigned to the CG_MOTION function. The control field of CG_MOTION is the area of the overlapping mesh that wraps around the pectoral fins. This setup allowed the pectoral fins to self-propel and kept the overlapping mesh in line with the pectoral fins at all times.

**Figure 2:**
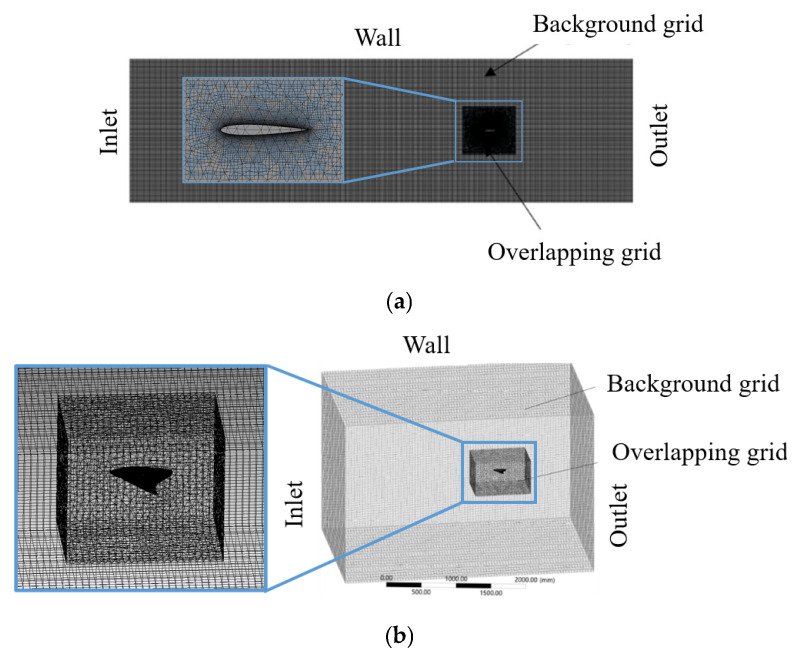
Schematic of the computational mesh and boundary conditions used in the simulation (**a**) for the 2D model; (**b**) for the 3D model.

The sizes of the computational domains were 8400 mm× 2400 mm for the 2D model and 2100 mm× 1200 mm× 600 mm for the 3D model. Their respective overlapping mesh sizes were 950 mm× 900 mm and 450 mm× 300 mm× 120 mm. Based on Rosenberger’s bionic observations [21], the selected kinematic parameters are listed in Table 1, and the meshes for calculating the flow field are shown in Figure 2.

**Figure 3:**
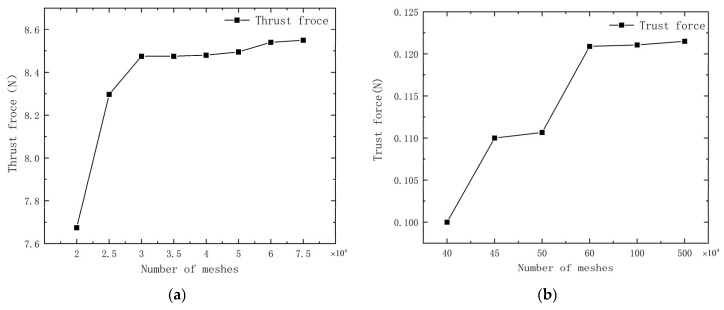

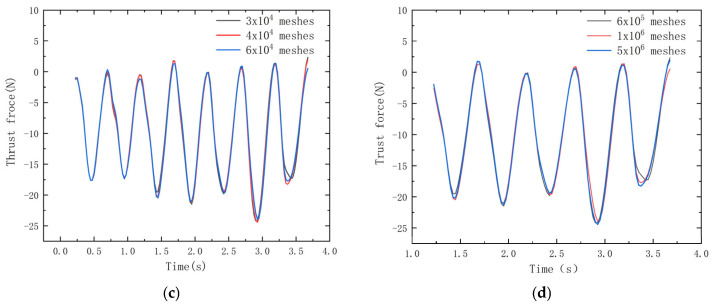
Mesh−independent validation of (**a**) average thrust curves for the 2D model with a different number of meshes; (**b**) average thrust curves for the 3D model with a different number of meshes; (**c**) thrust elongation graphs over time for 2D simulations with 30,000, 40,000, and 60,000 meshes; (**d**) thrust elongation graphs over time for 3D simulations with 600,000, 1,000,000, and 5,000,000 meshes.

The reliability of the computational method was verified by mesh-independent validation, and the mesh nodes were selected in the most appropriate arrangement. Next, mesh-independence was verified using different density meshes for both 2D and 3D models. The computational conditions for the 2D model were an incoming flow velocity of u=0 m/s, fluctuation frequency of f=1 Hz, amplitude of h=0.3 c, and wave number of 0.4. At the same time, the computational conditions for the 3D model were an incoming flow velocity of u=0 m/s, fluctuation frequency of f=1 Hz, amplitude of h=0.3 c, and wave number of 0.6. Figure 3a,b demonstrate the convergence of the thrust with increasing mesh density for both 2D and 3D simulations. Figure 3c,d reveal that the error in the average thrust for 2D simulations approximates 1% for mesh numbers of 30,000, 40,000, and 60,000, while for 3D simulations, the error is near 5% for mesh counts of 600,000, 1,000,000, and 5,000,000. Consequently, as the mesh count exerts a minimal influence on the overall simulation, the 2D simulations employ 40,000 meshes to economize on computational resources, and the 3D simulations employ 1,000,000 meshes.

## 3. Rationality Verification of 2D Simulation

Building upon the geometric modeling and numerical methods in Section 2, this section designed 2D simulations, 3D simulations, and experiments. Data from the 2D simulation and the 3D simulation, along with the experiment, were normalized. The rationality of the 2D simulation experiment was primarily assessed by comparing the effects of wave number, amplitude, and frequency on the propulsive force to determine whether the observed trends were consistent.

**Figure 4:**
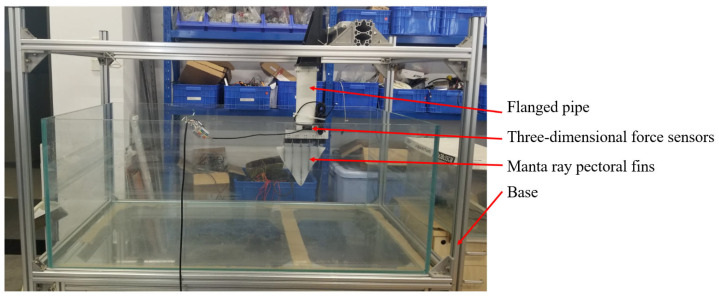
Physical diagram of the hydrostatic experimental device.

The hydrostatic test apparatus, depicted in Figure 4, comprised four principal components: the base, manta ray pectoral fins, sensors, and a flanged pipe. The base was constructed from aluminum profiles to support the large, ultra-white glass aquarium and to mount the pectoral fins of the manta rays being tested. Rubber pads were placed beneath the base to cushion its contact with the ground, mitigating potential platform vibrations. In order to minimize reflected waves from the walls, the ultra-white tanks used for the tests were approximately 7 times as wide as the measured pectoral fin chord length and 15 times as long as the pectoral fin chord length.

**Figure 5:**
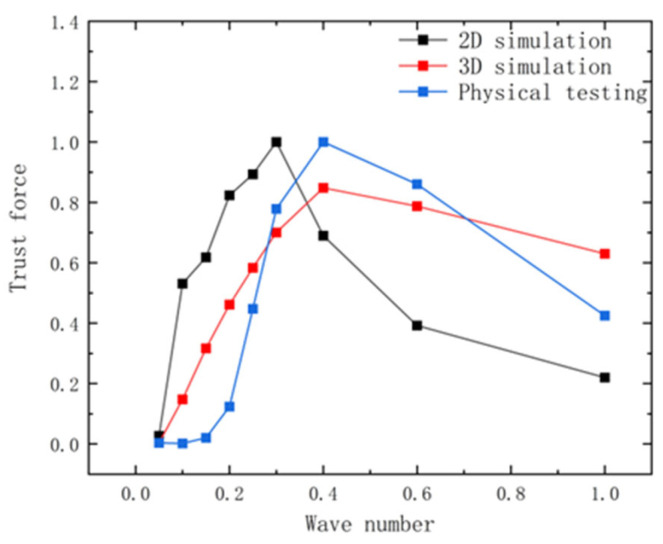
Comparison of thrust between experiment and simulation at different wave numbers after normalization.

The maximum magnitude for the 2D and 3D simulations, as well as the experiment, was set at 0.3 c, with a fluctuation frequency of 1.0 Hz for the experiment, and the subsequent data were normalized to facilitate comparison. From Figure 5, it can be seen that both simulated and experimental thrust values increase and then decrease with the rise in the wave number, and the maximum value of the thrust occurs in the range of a wave number of 0.3 to 0.6.

**Figure 6:**
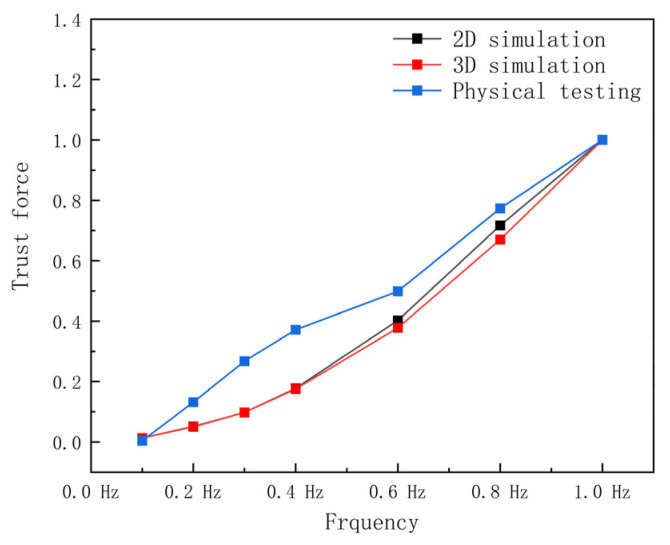
Comparison of thrust between experiment and simulation at different frequencies after normalization.

To further investigate the performance fluctuations of manta ray pectoral fins, the wave number was held constant at 0.4 waves for the experiment. Figure 6 presents the comparison of normalized pectoral fin thrust curves from simulations and experiments at various frequencies, demonstrating a strong correlation, particularly between the 2D and 3D simulations. With increasing frequency, the thrust curves from both the experiment and simulation exhibit an upward trend.

**Figure 7:**
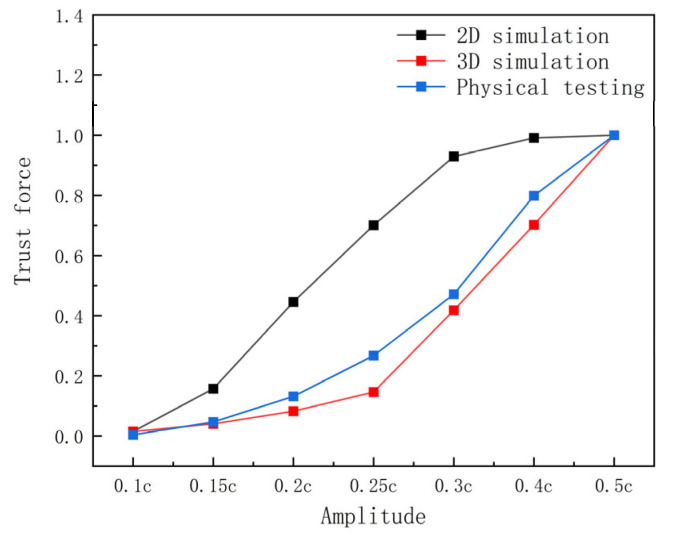
Comparison of thrust between experiment and simulation at different amplitudes after normalization.

Figure 7 illustrates that with increasing amplitude, the 2D simulation’s thrust curve rises sharply up to an amplitude of 0.3 c and then levels off, while the 3D simulation’s thrust curve demonstrates a slowly increase before 0.25 c and a steep increase after 0.25 c. This occurs because the 3D pectoral fins exhibit linear amplitude variation along the spreading direction, resulting in an average amplitude that is half the maximum amplitude and below the threshold for thrust decay.

In the real 3D pectoral fin motion, there is significant flow in the airfoil spreading direction (from the fin root to the fin tip or in the opposite direction), and the 3D pectoral fin deformation is distributed, with different motion modes and phase differences at different locations (proximal body side and fin tip). The 2D model only considers the flow in a single cross-section and ignores the lateral coupling effects. Therefore, the 2D model cannot completely capture the spatial distribution of the fluid during propulsion. However, it is the chordal flow, rather than the spreading direction, that significantly influences the hydrodynamic performance of manta rays. Consequently, although a 2D model has inherent limitations, it can still be used to investigate the hydrodynamic performance of these creatures.

The above analysis indicates that the hydrodynamic performance trends of manta rays’ pectoral fins in 2D and 3D simulations and experiments are broadly consistent, with the 2D simulations providing a closer reflection of the manta rays’ hydrodynamic patterns.

## 4. Study of Hydrodynamic Influences

### 4.1. Kinematic Modeling of Manta Ray Pectoral Fins

According to Russo et al. [22], the equations of motion of manta rays during fluctuating swimming are presented below:

(1) $$\theta (s,t)=\theta_{max}s\sin(\omega t-kx)$$

(2) k=2π/λ

(3) ω=2πf

where θ is the pectoral fin joint angle, $\theta_{max}$ is the maximum magnitude, *s* is the spread length, *t* is the movement time, λ is the traveling wave wavelength, and f is the traveling wave frequency.

**Figure 8:**
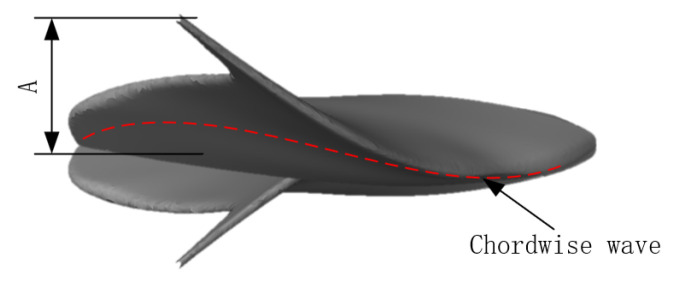
Schematic of manta ray movement.

The primary aim of this study was only to analyze the effect of kinematic parameters on manta rays when swimming in a straight line, neglecting subtle displacements in the x- and y-directions. Consequently, the equations of motion were formulated only for significant fluctuations in the z-direction. We assumed a constant chordwise amplitude and modeled the spreading amplitude as a primary curve. After substituting the chord length, we described the pectoral fin’s fluctuations as follows:

(4) z=A(y)sin(kx−ωt)

where *A*(*y*) is the amplitude control factor with respect to the y-coordinate. As shown in Figure 8, *A* is the maximum magnitude of pectoral fin spread.

In order to evaluate the propulsive force and the efficiency of propulsion, we calculated the pressure and total energy consumption of the manta ray’s pectoral fins. The relationship is defined as

(5) $$F_{P}(t)=\int_{As}pn_{i}dA$$

(6) $$P=\frac{1}{T}(\int_{0}^{T}A(y)\cos(kx-\omega t)\cdot F_{P}(t)dt)$$

(7) $$A_{s}=cs$$

where $F_{p}(t)$ is the pressure, p is the pressure, $n_{i}$ represents the unit normal vector of the *i*th surface and is oriented vertically outward from the control surface, and $A_{s}$ is the surface area of the pectoral fin. The term to the right of the middle sign in the equation is the average of the integral of the product of normal pressure and velocity on the pectoral fin surface over the 0–T moments.

As discussed in Maertens et al. [23] (2015), the Froude advancing efficiency is not appropriate in this context. Therefore, the quasi-propulsive efficiency $\eta_{QP}$ [24] was employed, which was compared with the useful power (the drag *R* of the stiffened towed body at the same speed multiplied by the speed U), as follows:

(8) $$\eta_{QP}=\frac{RU}{P}$$

The energy coefficient [23] is the ratio of power consumption to speed, and in general, a smaller energy coefficient means that less energy is consumed per unit of velocity gained. The energy coefficient can be defined as

(9) $$C_{P}=\frac{P}{\frac{1}{2}\rho A_{S}U^{3}}$$

where P is the pressure, and ρ is the fluid density.

### 4.2. Effect of Wave Number on Pectoral Fin Hydrodynamic Performance

In this section, we used a one-factor analysis to investigate the impact of wave number on the steady-state swimming performance of manta ray pectoral fins, with the fluctuation frequency taking the value of 1 Hz, the amplitude taking the value of 0.3 c, and the interval of the wave number varying from 0.05 to 1.0. Then, we assessed the influence of wave number on the hydrodynamic performance of manta ray pectoral fins by examining the effects of varying wave numbers on mean thrust, mean lift, and efficiency during fluctuations in the absence of incoming flow velocity.

**Figure 9:**
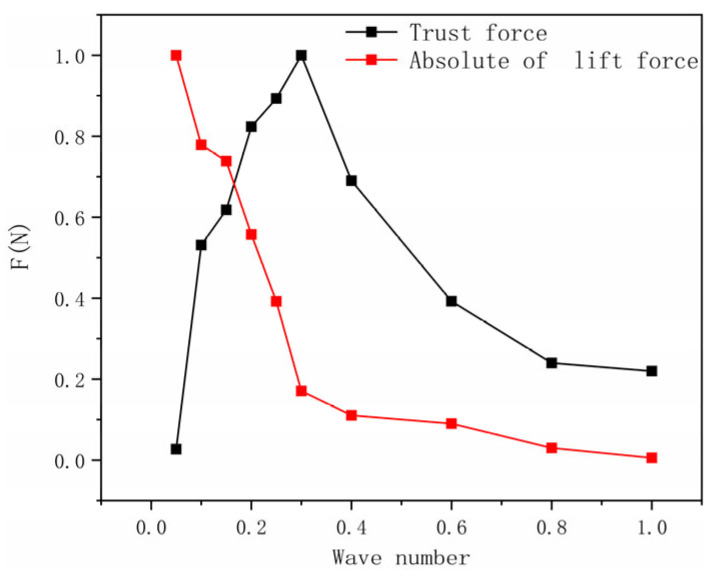
The normalized plot of the relationship between thrust, lift, and wave number.

As the wave number increases, the overall trend in thrust initially rises before gradually declining, as illustrated in Figure 9. Thrust values are minimal at both low and high wave numbers, reaching a peak between 0.2 and 0.4 wave numbers, and then decreasing rapidly from 0.4 to 1.0 wave numbers. Concurrently, the average lift exhibits a decreasing trend, with a significant reduction occurring between 0.2 and 0.4 wave numbers; this decline becomes more moderate when the wave number exceeds 0.6. Analysis suggests that the lateral perturbation of water by manta ray pectoral fins is more pronounced at lower wave numbers. As shown in Figure 9, the movements of the pectoral fins during this range are predominantly vertical, which limits their ability to generate effective thrust.

**Figure 10:**
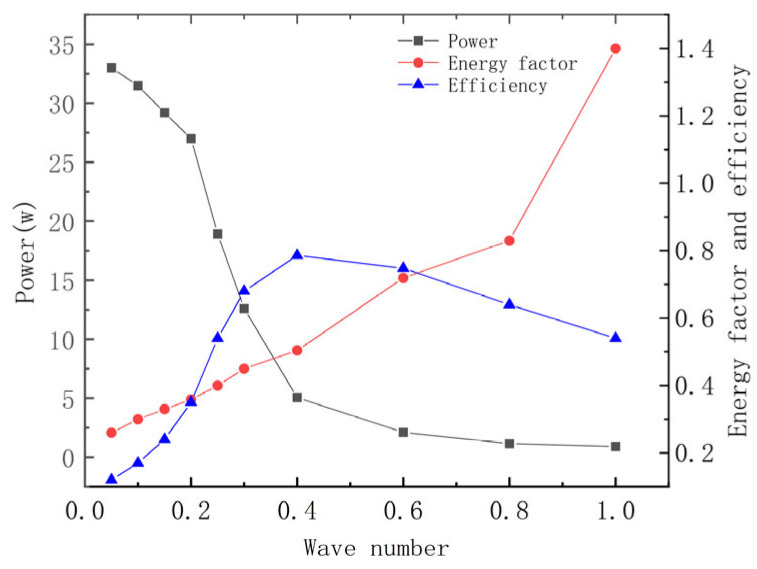
Comprehensive energy consumption graph.

Figure 10 demonstrates that total energy consumption decreases as the wave number increases, while the energy coefficient exhibits an opposite trend, increasing overall. Furthermore, the reduction in power consumption between 0.2 and 0.4 wave numbers is particularly steep, whereas the decline in power consumption begins to level off after 0.4 wave numbers. Efficiency tends to increase before subsequently decreasing, with a peak occurring at 0.4 wave numbers, aligning with Rosenberger’s findings on manta rays in bionics.

**Figure 11:**
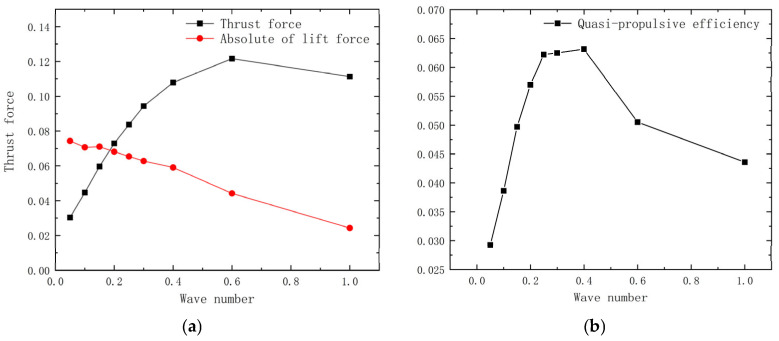
Simulation results at different wave numbers. (**a**) Propulsion and lift; (**b**) quasi-propulsive efficiency.

Figure 11a illustrates that the lift decreases with an increasing wave number, reaching a minimum at a 1.0 wave number. This behavior occurs because, as the wave number decreases, the pectoral fins behave more like a flat plate oscillating vertically, which gradually enhances the component of force in the lift direction. At this point, the force component is significant in the direction perpendicular to the pectoral fins and minimal in the forward direction. The fins generate thrust primarily through vortex shedding at their posterior edges, a process strongly influenced by fluctuations at these edges. As the wave number increases, the chord length of the pectoral fins responsible for vortex shedding shortens, resulting in reduced thrust. As shown in Figure 11b, the efficiency initially increases, reaches a peak at approximately 0.4 wave number, and then declines, indicating that the maximum quasi-propulsive efficiency occurs at this value.

### 4.3. Effect of Frequency and Amplitude on Pectoral Fin Hydrodynamic Performance

Based on the results in Section 4.2, a wave number of 0.4 yields greater propulsive force and quasi-propulsive efficiency. Consequently, the wave number was fixed at 0.4, and experiments were conducted to identify the optimal pairing of kinematic parameters, specifically frequency and amplitude. In these experiments, the amplitude ranged from 0.1 c to 0.5 c, and the frequency ranged from 0.4 Hz to 1.0 Hz, totaling 35 test cases.

**Figure 12:**
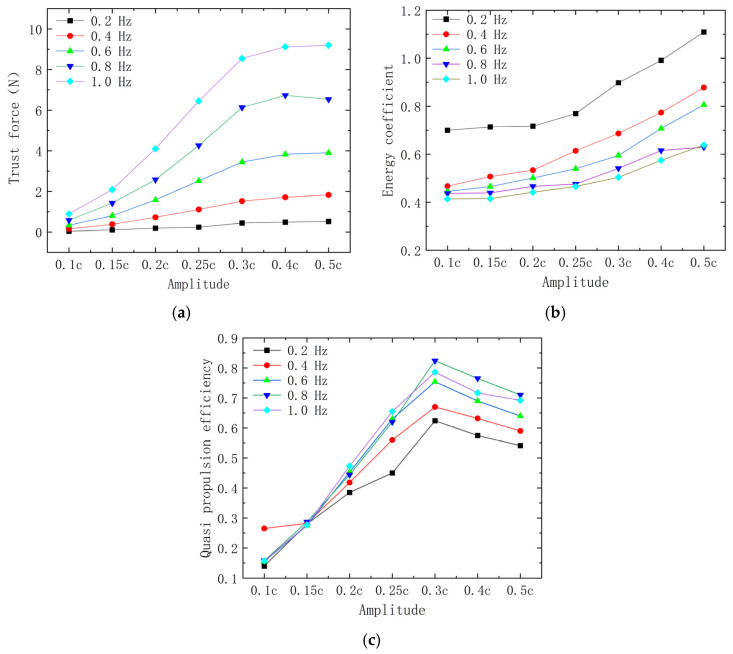
Hydrodynamic performance obtained for different parameters. (**a**) Thrust; (**b**) energy coefficient $c_{p}$; (**c**) quasi-propulsive efficiency $\eta_{Qp}$.

Figure 12 illustrates the trends in thrust, energy coefficient, and efficiency generated by manta ray pectoral fins at a wave number of 0.4. The results shown in Figure 12a indicate that as the fluctuation frequency increases, the thrust force also increases. Additionally, higher amplitudes contribute to greater thrust. For amplitudes below 0.3 c, the thrust increases rapidly with amplitude; however, beyond 0.3 c, the rate of increase begins to plateau. As the frequency of pectoral fin beats increases, the rate of fluid acceleration due to periodic motion is enhanced, leading to a significant increase in thrust. A larger amplitude results in a greater volume of water being displaced by the pectoral fins during oscillation, which in turn pushes more fluid backward and generates a larger reaction force. However, when the amplitude exceeds 0.3 c, the increase in thrust levels off. This phenomenon may be attributed to the rise in nonlinear fluid drag and alterations in the tail vortex shedding pattern, whereby the positive effects of increased amplitude are gradually counterbalanced by the additional drag.

The results presented in Figure 12b demonstrate a decreasing energy coefficient as frequency increases. This indicates that, at higher frequencies, pectoral fin fluctuations can achieve greater speeds while consuming the same amount of energy. Notably, the energy coefficient tends to increase with amplitude, particularly after the amplitude exceeds 0.3 c, where the increase becomes more pronounced. Consequently, a higher amplitude does not enhance energy utilization. Observations from bionomics indicate that the average amplitude of ray-finned fish is approximately 0.32 c, which supports the credibility of the simulation presented here. In the figure, $c_{p}$ shows an accelerating trend with increasing amplitude and a decreasing trend with increasing frequency, so it can be assumed that using low-amplitude and high-frequency propulsion has a better energy utilization.

Figure 12c illustrates that the quasi-propulsive efficiency of the pectoral fins is greater at higher frequencies. However, for a constant amplitude, the variation in quasi-propulsive efficiencies across different frequencies is minimal. In contrast, the amplitude plays a significant role in the change in efficiency. As amplitude increases, efficiency initially rises before subsequently decreasing, with the peak quasi-propulsive efficiency occurring around 0.3 c, consistent with the motion dynamics of manta rays. As the amplitude increases, efficiency initially rises, because thrust increases at a faster rate than energy consumption. However, beyond an amplitude of 0.3 c, the rate of energy consumption’s growth surpasses that of thrust’s growth, leading to a decline in efficiency. The increase in quasi-propulsive efficiency at higher frequencies can be attributed to the enhanced formation of a continuously ordered tail vortex structure (e.g., an anti-Kármán vortex street), which improves propulsive stability and optimizes the utilization of fluid kinetic energy.

### 4.4. Time Cost Comparison

**Figure 13:**
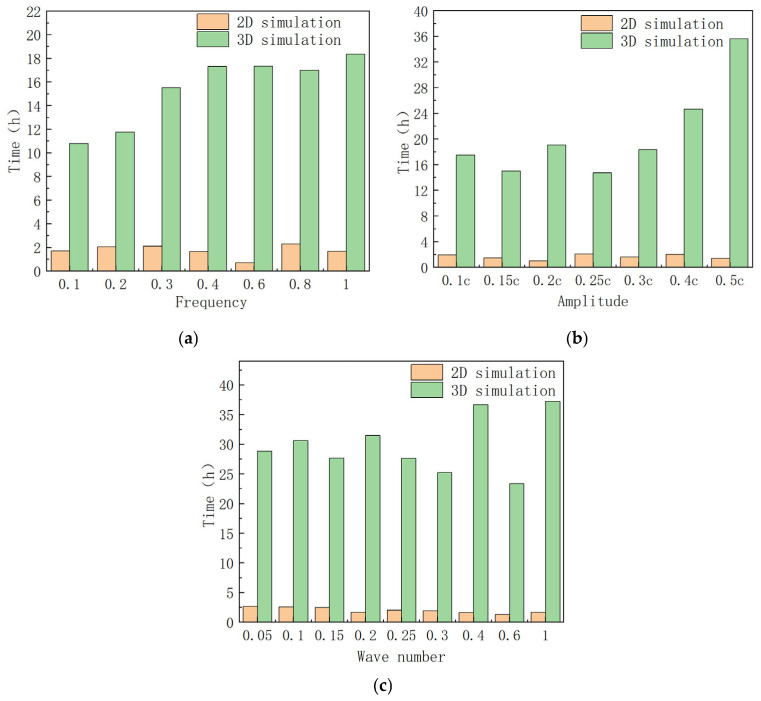
Time spent in 2D simulation and 3D simulation with different parameters. (**a**) Frequency; (**b**) amplitude; (**c**) wave number.

Due to the significantly lower number of meshes in 2D simulations compared to 3D simulations, 2D simulations offer a computational advantage in terms of time efficiency. The simulation times for both 2D and 3D simulations were recorded across various frequencies, amplitudes, and wave numbers to generate the time cost comparison graph presented in Figure 13.

As illustrated in Figure 13, the 2D simulation reduces the average simulation time by approximately 78.8% compared to the 3D simulation across the different frequencies, amplitudes, and wave numbers examined. Consequently, employing 2D simulations to investigate the hydrodynamic performance of manta ray pectoral fins can significantly enhance the efficiency of the research.

## 5. Research on Propulsion and Propulsion Efficiency

Based on the previous section, optimal thrust and quasi-propulsive efficiency are achieved at frequencies between 0.6 Hz and 1.0 Hz, amplitudes from 0.25 c to 0.325 c, and wave numbers ranging from 0.35 to 0.6. Consequently, the analysis focused on these parameter ranges: frequency (0.6–1.0 Hz), amplitude (0.25 c–0.325 c), and wave number (0.35–0.6). The factor levels are summarized in Table 2. A total of 80 experimental runs were conducted using a mixed orthogonal design.

The visual analysis indicates that the wave number has the most significant influence on speed, power consumption, and energy efficiency, while fluctuations in the parameters most significantly affect quasi-propulsive efficiency in Table 3.

**Figure 14:**
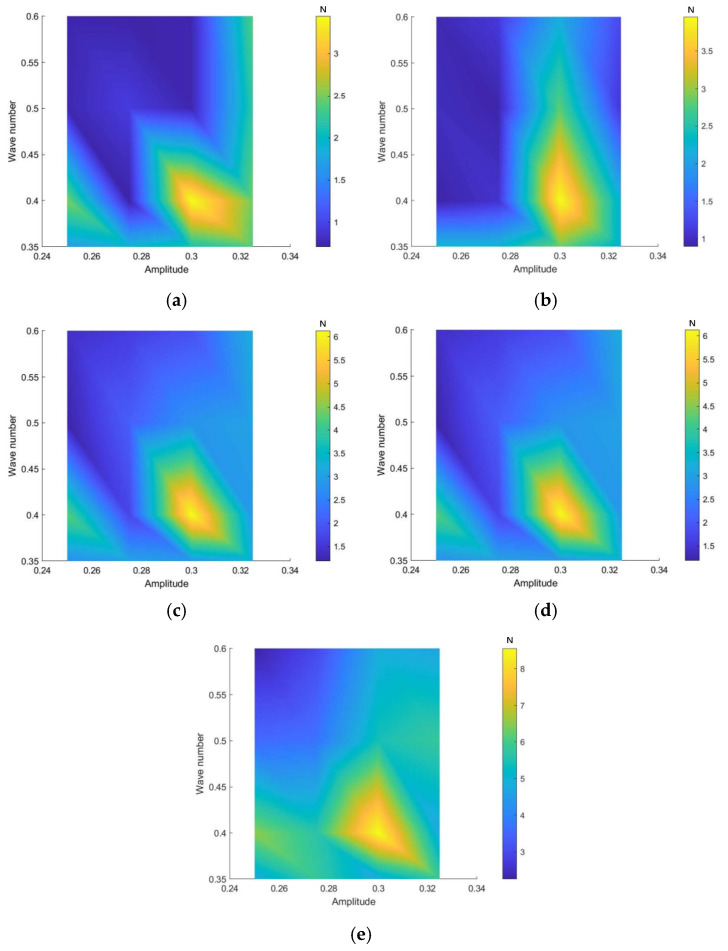
Effect of wave number and amplitude on the swimming thrust of manta rays at different frequencies: (**a**) 0.6 Hz; (**b**) 0.7 Hz; (**c**) 0.8 Hz; (**d**) 0.9 Hz; (**e**) 1.0 Hz.

It can be summarized from Figure 14 that the thrust generated by manta rays increases with frequency for a given amplitude and wave number, and for each frequency, the maximum thrust occurs at a wave number of 0.35–0.4 and amplitude of 0.275 c–0.325 c. Additionally, it is easier to modify thrust by varying frequency rather than wave number or amplitude. The thrust exceeds 3.04 N when the wave number is between 0.35 and 0.4, the frequency is between 0.7 and 1 Hz, and the amplitude is between 0.275 c and 0.325 c. The thrust reaches its peak value of 8.55 N at a frequency of 1 Hz, an amplitude of 0.3 c, and a wave number of 0.4.

**Figure 15:**
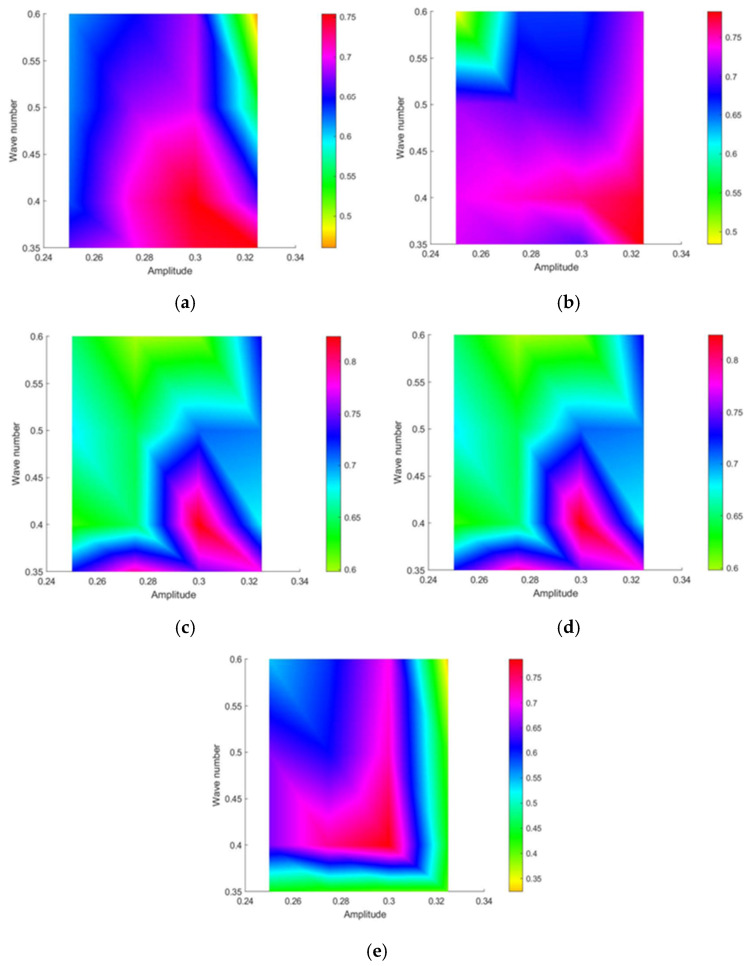
Effect of wave number and amplitude at different frequencies on the quasi-propulsive efficiency of manta rays’ swimming: (**a**) 0.6 Hz; (**b**) 0.7 Hz; (**c**) 0.8 Hz; (**d**) 0.9 Hz; (**e**) 1.0 Hz.

In Figure 15, it can be observed that quasi-propulsive efficiency is minimally affected by frequency but is predominantly influenced by wave number and amplitude, with amplitude having the most significant impact. The maximum value of the quasi-propulsive efficiency occurs at a wave number of 0.35–0.45 and amplitude of 0.275 c–0.325 c. The quasi-propulsive efficiency exceeds 70.4% at a wave number of 0.35–0.4, frequency of 0.7–0.9 Hz, and amplitude of 0.275 c–0.325 c. Furthermore, the quasi-propulsive efficiency reaches a maximum value of 82.4% when the frequency is 0.8 Hz, the amplitude is 0.3 c, and the wave number is 0.4.

In practical applications, it is desirable for manta rays to achieve both a high propulsive force and high quasi-propulsive efficiency during swimming. Based on these conclusions, the quasi-propulsive efficiency is 78.6% when the thrust is maximized, and the thrust is 6.13 N when the quasi-propulsive efficiency is maximized. In other words, maximum propulsive force and maximum quasi-propulsive efficiency cannot be satisfied simultaneously. Further analyzing Figure 14 and Figure 15, we can summarize that a large thrust and quasi-propulsive efficiency can be obtained at a frequency in the range of 0.7–0.9 Hz, an amplitude in the range of 0.3 c–0.325 c, and a wave number in the range of 0.35–0.4.

## 6. Conclusions

In this study, we numerically simulated the motion parameters of manta ray pectoral fins across various wave numbers, amplitudes, and frequencies. We obtained graphs depicting thrust, lift, power consumption, energy coefficients, and quasi-propulsive efficiencies, complemented by validation analyses from three-dimensional simulations and experimental data. The following conclusions were drawn:

Firstly, we designed simulation experiments to investigate the hydrodynamic performance of 3D manta ray pectoral fins and constructed a hydrodynamic testing platform to conduct relevant experiments. Then, we used the normalization method to compare the hydrodynamic performance trends of 2D simulation, 3D simulation, and experiments, and the results showed that the trends of the three were basically consistent, which proved the reasonableness of using 2D simulation to explore the pectoral fin swimming pattern of manta rays. Additionally, a comparison of time costs demonstrated that research efforts could be made more efficient using two-dimensional models.

Secondly, among the three kinematic parameters, amplitude, frequency, and wave number exert the most significant influence on velocity, power consumption, and energy efficiency, and the amplitude has the highest impact on quasi-propulsive efficiency. The thrust and quasi-propulsive efficiency initially increase with an increasing wave number, then gradually decrease. When the frequency is increased at a fixed wave number, the thrust continues to rise. For amplitudes below 0.3 c, thrust tends to increase with amplitude, reaching a maximum at 0.3 c, after which it plateaus. Similarly, quasi-propulsive efficiency increases with amplitude at a fixed number of waves and frequency, peaking at 0.3 c. Overall, these results suggest that manta ray pectoral fins achieve better energy utilization through low-amplitude, high-frequency propulsion optimized at specific wave numbers.

Third, orthogonal experimental analysis indicates that manta rays can generate greater thrust and quasi-propulsive efficiency at wave numbers between 0.35 and 0.4. Specifically, the quasi-propulsive efficiency exceeds 70.4% at frequencies of 0.7–0.9 Hz and amplitudes of 0.25 c–0.325 c. Thrust forces greater than 3.04 N are observed at frequencies between 0.7 and 1 Hz and amplitudes from 0.275 c to 0.325 c. The combination of frequencies between 0.7 and 0.9 Hz and amplitudes between 0.3 c and 0.325 c yields both a high thrust and quasi-propulsive efficiency.

Manta rays achieve efficient propulsion through the dynamic movements of their pectoral fins, where the combinations of amplitude, wave number, and frequency directly influence hydrodynamic performance. Investigating these parameters is essential for understanding the mechanisms underlying biological motion, which can elucidate how manta rays attain low-energy, high-mobility locomotion through the synergistic optimization of morphology and movement. Furthermore, these insights enhance the field of bionic hydrodynamics and offer significant design principles for biomimetic engineering. The findings provide a theoretical foundation for developing high-performance bionic manta ray robots, which have diverse applications in areas such as ocean engineering.

## Figures and Tables

**Table 1:** Movement parameters of manta rays.

Motion Parameters	Symbols	Value
Motion frequency (Hz)	f	0.2, 0.4, 0.6, 0.8 and 1.0
Motion amplitude (c)	A	0.1, 0.15, 0.2, 0.25 and 0.3
Wave number	W	0.2, 0.4, 0.6, 0.8 and 1.0

**Table 2:** Factor level table.

	Factor		
Level	Frequency (Hz)	Amplitude (c)	Wave Number
1	0.6	0.25	0.35
2	0.7	0.275	0.4
3	0.8	0.3	0.5
4	0.9	0.325	0.6
5	1.0	-	-

**Table 3:** Visual analysis table.

	Frequency	Amplitude	Wave Number
Velocity range	0.314	0.138	0.384
Quasi-promotional efficiency range	0.102	0.411	0.304
Power consumption range	4.341	2.937	6.647
Energy factor range	0.296	0.304	0.74

## Data Availability

The study data can be obtained by email request to the authors.
